# Missegregation of Chromosome 3 and Generation of Monosomy 3 in the Proliferating Uveal Melanoma Cells Under Hyperglycemia

**DOI:** 10.1167/iovs.66.15.10

**Published:** 2025-12-02

**Authors:** Aysegül Tura, Svenja R. Sonntag, Nikolas Christian Cornelius von Bubnoff, Malte Spielmann, Salvatore Grisanti

**Affiliations:** 1 Department of Ophthalmology, University Clinic Schleswig-Holstein (UKSH), University of Lübeck, Lübeck, Germany; 2 Department of Hematology and Oncology, Medical Center, University Hospital Schleswig-Holstein, Campus Lübeck, Lübeck, Germany; 3 Institute of Human Genetics, University Hospitals Schleswig-Holstein, University of Lübeck and University of Kiel, Lübeck, Germany

**Keywords:** uveal melanoma, monosomy 3, hyperglycemia, nucleolus, chromosome missegregation

## Abstract

**Purpose:**

In uveal melanoma (UM), coexistence of the fatal monosomy 3 with the benign gain of chromosome 6p occurs rarely. The spatial organization of chromosomes can be influenced by the nucleoli, which become larger under hyperglycemia. We therefore hypothesized that hyperglycemia may be responsible for chromosome-specific aberrations in UM and analyzed its effect on nucleolar organization, chromosome territories, and missegregation rates in vitro.

**Methods:**

UM cell lines 92.1 and OMM2.5, UM cells from the primary tumors of two patients, and Tenon fibroblasts from a control were incubated in normo- or hyperglycemic medium (with 5.5 or 25 mM glucose, respectively) for one day, followed by the mitotic arrest with Nocodazole for 18-24 hours and recovery in fresh medium. Co-detection of proteins with the centromeres of chromosomes 3 and 6 was performed by two-dimensional immunofluorescent in situ hybridization.

**Results:**

In the UM cells undergoing interphase, hyperglycemia promoted the dislocation of chromosome 3 toward the center along with nucleolar growth. During prometaphase, the mean angle between the centromeres of chromosome 3 was reduced below 90° under hyperglycemia (*P* = 0.02). During the later mitotic phases, hyperglycemia resulted in a 3.8-fold increase in the missegregation rate of chromosome 3 in UM cells (*P* < 0.001), whereas chromosome 6 rather than 3 was more prone to missegregation in the normoglycemic UM cells and hyperglycemic Tenon fibroblasts.

**Conclusions:**

Hyperglycemia can favor chromosome-specific aneuploidies by altering chromosome territories in a cell-type dependent manner. Prevention of hyperglycemia may be a simple therapeutic approach to impede the generation of monosomy 3 in UM.

Despite the successful local control of uveal melanoma (UM), almost half of the patients sadly develop fatal metastases mainly in the liver.^1^^–^^6^ The risk of metastasis is particularly high for the tumors with the loss of one copy of chromosome 3 (monosomy 3),^1^^–^^6^ which is considered to be an early event in the course of UM.^1^^,^^2^^,^^6^ However, the acquisition of monosomy 3 can also occur later,^7^ which may be promoting the faster or unexpected growth of primary tumors.^7^^,^^8^ Likewise, the presence of monosomy 3 in the metastasized UM cells was associated with a rapid disease progression and worse survival rate.^9^ In contrast, the gain of chromosome 6p, which occurs in an almost mutually exclusive pattern with monosomy 3, is associated with a good prognosis.^1^^–^^6^ This peculiar pattern indicates a molecular switch that directs the proliferating UM cells either towards a malignant or benign genotype by unknown mechanisms.^1^^,^^3^^,^^4^^,^^6^ Understanding the cause for the chromosome-specific aberrations in UM may be therefore crucial for patients’ life.

A recent study reported that human chromosomes 3, 6, and X are particularly prone to missegregation because of their large size and smaller centromere.^10^ Although this finding may provide an explanation for the frequent copy number alterations of chromosomes 3 and 6 in UM,^1^^–^^6^ it is not sufficient to explain the mutually-exclusive pattern. Because the loss of one chromosome would generate space for another chromosome, coexistence of 6p gain and monosomy 3 should indeed be the logical consequence of the chromosomal missegregation. Because this is not the fact, further non-random events during the malignant transformation of UM cells seem to be causative.

The chromosomes are indeed not randomly distributed in the nucleus. Chromosomes tend to occupy distinct territories, which can change depending on the cellular demands, with the activated genes usually being relocated from the nuclear periphery towards the center.^11^ The spatial organization of chromosomes can be further influenced by the nucleoli, which can form a physical barrier in the nucleus, keeping certain chromosomal domains separated.^12^^,^^13^ It is general knowledge that larger nucleoli are associated with a worse prognosis in UM.^4^ Likewise, the hypertrophy of nucleoli serves as a reliable indicator of malignancy in diverse tumors.^14^^,^^15^ The undifferentiated cells also tend to have one large and hyperactive nucleolus instead of several smaller nucleoli.^15^ However, it remains unknown, why the nucleoli become enlarged in some UMs and whether the oversized nucleoli can alter the chromosome territories in UM. We theoretized that large nucleoli may create a physical barrier, which may promote the missegregation of chromosome 3 rather than chromosome 6p and prevent the coexistence of these anomalies in the mitotic UM cells.

The nucleoli are membrane-free and dynamic structures in the nucleus, with the major function being the synthesis of ribosomes, which is the most energy-consuming process in a cell.^14^ Accordingly, the nucleolar activity is tightly regulated by the metabolic status of a cell, which in turn influences the size and morphology of nucleoli.^14^^,^^15^ For instance, a decrease in nucleolar size was detected in the muscle biopsies of elderly individuals who reduced their calorie intake modestly and increased their exercise.^16^ In contrast, the enlargement of nucleoli was observed in human lymphocytes under hyperglycemia.^17^ Remarkably, a hyperglycemic blood profile was reported in a recent study with 111 UM patients, with an average fasting glucose concentration of 117.71 mg/dL and standard deviation of 53.17 mg/dL,^18^ suggesting that some UM patients already fall into the prediabetic or diabetic range (fasting glucose between 100-125 mg/dL or above, respectively^19^^,^^20^) within six months of their tumor diagnosis.^18^ A subtle form of hyperglycemia was also noticed in a different cohort of non-diabetic UM patients compared to the age-matched controls, with a slight but significant increase in the fasting blood glucose levels together with an insulin-resistant serum profile in the former group.^21^ Insulin resistance is a pathophysiological condition which necessitates a higher level of insulin secretion for the maintenance of circulating glucose levels in the normal range.^22^^,^^23^ Insulin resistance is therefore associated with a longer period of postprandial hyperglycemia,^23^ which may remain undetected for many years due to the appearance of the fasting blood glucose concentration at physiological levels.^21^^,^^23^ Insulin resistance may also be creating a more hyperglycemic and permissive microenvironment for the hepatic micrometastases of UM by causing the excessive release of glucose from the liver, which serves as the major glucose reserve in the body.^22^ Accordingly, type 2 diabetes was associated with an increased risk of metastasis in a Swedish cohort of UM patients.^24^ Likewise, the unexpected growth of a primary UM was reported in a patient three to four years after her therapies for diabetic retinopathy, possibly because of the late transformation of some UM cells to the monosomy 3 genotype, as demonstrated by the presence of monosomy 3 cells in the tumor apex, but not the base.^7^ Moreover, three further case reports involved diabetic UM patients who either had metastases in multiple organs at initial presentation^25^ or who developed metastases within a relatively short period.^26^^,^^27^ However, insulin resistance and hyperglycemia are not included among the established prognostic factors for UM yet. Additionally, there are no studies so far investigating the effect of hyperglycemia on nucleoli, chromosome territories, and chromosomal missegregation in UM.

To elucidate these aspects, we incubated the UM cell lines 92.1 and OMM2.5, as well as the cultures derived from the primary tumors of two UM patients under normo- versus hyperglycemia and enriched the cells in different mitotic phases using nocodazole, which can reversibly interfere with the polymerization of microtubules.^28^ The human Tenon fibroblasts were treated in the same pattern as control. The organization of nucleoli during distinct cell cycle phases was evaluated by the expression of the nucleolar protein Ki67.^29^ The missegregation rates of chromosomes 3 and 6 were detected in the mitotic cells that were tetraploid for these chromosomes. The positions of chromosomes 3 and 6 with respect to the nuclear center and nucleolar domains were also analyzed during different cell cycle stages. The post-mitotic fates of UM cells were also evaluated by analyzing the copy numbers of chromosomes 3 and 6 in the interphase nuclei after culturing the cells for one week under normo- versus hyperglycemia.

## Methods

### Cell Culture

The UM cell lines 92.1 and OMM2.5, which have been characterized extensively,^30^^–^^32^ were kindly provided by Prof. Martine J. Jager (Leiden University Medical Center, Leiden, The Netherlands). The 92.1 cells originate from the primary tumor of a female UM patient,^30^^–^^32^ whereas the OMM2.5 cell line was established from the liver metastases of a male patient.^30^^,^^32^ Both cell lines were authenticated by the profiling of short tandem repeats.^30^

Cultures of the primary UMs (UM_f81 and UM_m60) were established from the enucleation samples of two patients who were operated at the University Eye Clinic Lübeck, Germany. The UM_f81 cells were generated from a female patient (age at diagnosis: 81 years; T3aN0M0) who underwent primary enucleation, whereas the UM_m60 cells originated from the irradiated tumor of a male patient (age at diagnosis: 60 years; T3aN0M0). Tumor classification was performed based on the eighth edition of the staging manual of American Joint Committee on Cancer.^33^ Approximately one half of each tumor sample was processed for the cultivation of UM cells within one hour of tissue collection as described.^34^ The remaining tissues were fixed in formalin, embedded in paraffin, and processed for hematoxylin-eosin staining and BAP1-immunohistochemistry for tumor characterization (Supplementary Fig. S1a). UM cells in the passages 2 through 7 were used for the subsequent experiments.

Cultures of human Tenon fibroblasts (HTF) were generated as described^35^ within one hour of tissue collection from a 67-year old female patient with no known history of ocular tumors and who underwent cataract surgery at the University Eye Clinic Lübeck, Germany. The Tenon fibroblasts that were in the passages 3 through 7 were used for the experiments.

The analysis of human samples was approved by the local ethic committee of the University of Lübeck (File number: 10–200) and the study conforms to the guidelines of the Declaration of Helsinki as revised in Tokyo and Venice. The patients received an explanation about the nature and possible consequences of the study and gave informed consent before their inclusion. The human tissue experiments complied with the guidelines of the ARVO Best Practices for Using Human Eye Tissue in Research (Nov2021). The patients’ data were retrieved from their medical records.

All cells were grown under normoxic conditions at 37°C with 5% CO_2_ in human plasma like medium (HPLM) supplemented with 10% fetal bovine serum, 2 mM L-glutamine, 100 units/mL penicillin, and 100 µg/mL streptomycin (Thermo Fisher Scientific, Waltham, MA, USA) and passaged by trypsinization. HPLM is a recently formulated medium with a glucose concentration of 5 mM.^36^ After supplementation with 10% serum, the final glucose concentration of the culture medium reaches approximately 5.5 mM, which is considered as normoglycemic.^37^ The cultures of UM cells and HTF were characterized by the expression pattern of fibroblastic and melanocytic markers as detected by immunocytochemistry (Supplementary Fig. S1b).

### Culturing the Cells Under Normo- Versus Hyperglycemia

Cells that were seeded into 8-well slides or 6-well plates were allowed to attach overnight. The medium was replaced on the next day with normo- or hyperglycemic HPLM, with the latter being supplemented with a final glucose concentration of 25 mM^37^ and the cells were incubated for one day. Cells in the six-well plates were dissociated by trypsinization and processed as cytospins by centrifuging onto Superfrost slides at 130 g for five minutes. Cells in the eight-well slides were used for fluorescent immunocytochemistry as described below.

### Enrichment of Cells in Different Mitotic Stages

Cells in six-well plates were cultured under normo- or hyperglycemia for one day as described above. Afterward, nocodazole (Sigma-Aldrich Corp., St. Louis, MO, USA) was added into the test wells at a final concentration of 1 µg/mL and the cells were incubated further for 18 to 24 hours. The culture medium was then removed and the cells were rinsed briefly in PBS. The cells were replenished with fresh normo- or hyperglycemic medium without nocodazole, incubated for 15 to 240 minutes with 15-minute intervals, and processed as cytospins. The optimal time point for the enrichment of cells in distinct mitotic phases was determined individually for each cell type and culture condition after the nocodazole-washout for 15-minute intervals that amounted up to 240 minutes. Under hyperglycemia, all the tested cells could recover faster from the mitotic arrest and proceed approximately 15 minutes earlier into each mitotic stage compared to the normoglycemic cells.

### Immunohistochemistry and Fluorescent-Immunocytochemistry

Immunohistochemistry was performed on the paraffin sections of the tumors as described^34^ using the primary antibodies against BAP1 (rabbit, ab199396, 1:20 dilution; Abcam, Cambridge, UK) followed by horseradish peroxidase–conjugated anti-rabbit secondary antibodies (UK, 111-035-003; 1:250 dilution; Jackson ImmunoResearch Labs, West Grove, PA, USA) and the horseradish peroxidase–green chromogen (42 Life Sciences, Bremerhaven, Germany). The nuclei were counterstained with nuclear fast red.^34^ Images of the entire tumor area were acquired under 200× magnification by light microscopy (Leica, Wetzlar, Germany).

Immunocytochemistry was performed as described^34^ using the primary rabbit antibodies against BAP1 (1:100; Abcam), fibroblast specific protein 1/S100A4 (16105-1-AP, 1:200 dilution; Proteintech, Planegg-Martinsried, Germany), Melan-A (ab210546; 1:100 dilution; Abcam), or MITF (AB4139, 1:200 dilution; Merck, Darmstadt, Germany), followed by the Cy3-conjugated anti-rabbit secondary antibodies (111-165-003; 1:400 dilution; Jackson Immunoresearch). The nuclei were counterstained with 0.5 µg/mL 4′,6-diamidino-2-phenylindole (DAPI) in PBS for 10 minutes. Images were acquired under 200× magnification using a fluorescence microscope (DMI 6000B; Leica) with the following filter sets: A4: Excitation (Ex): 360/40, Emission (Em): 470/40 nanometers (nm); Y3: Ex: 545/30, Em: 510/75 nm. Quantification of nuclear BAP1 immunoreactivity in non-overlapping nuclei from three independent experiments was performed by the automated selection via threshold adjustment on the nuclear staining channel and redirecting the measurements of mean intensity and integrated density to the BAP1 immunostaining channel via the Fiji software.^38^ The background values were removed by subtracting the average BAP1 intensity of the negative control. The number of quantified cells per group in each experiment ranged between 403–616 and 467–906 for the 92.1 and OMM2.5 cell line, respectively.

### Two-Dimensional Immuno-FISH

Detection of chromosomes 3 and 6 together with the proteins of interest was performed by two-dimensional Immuno-FISH on the cytopins of cultured cells as described,^39^ using the primary antibodies against alpha-tubulin (clone DM1A, 05-829, 1:500 dilution; Merck) or Ki67 (ab15580; 1:500 dilution; Abcam), followed by the Alexa 488-conjugated anti-mouse (A-11001; 1:100 dilution; ThermoFisher, St. Louis, MO, USA) or Cy5-conjugated anti-rabbit secondary antibodies (111-175-144, 1:50 dilution; Jackson Immunoresearch). The centromeric probes with Spectrum-Orange or -Aqua conjugations were applied for labeling the chromosomes 3 and 6, respectively (Abbott GmbH, Wiesbaden, Germany). Because the z-axis of cells becomes negligible after cytocentrifugation, FISH on cytospins is considered to be two-dimensional.^40^ Images were acquired under 400× magnification using a fluorescence microscope (DMI 6000B; Leica) with the abovementioned filter sets A4 and Y3, as well as the CFP (Ex: 436/20, Em: 480/40 nm), L5 (Ex: 480/40, Em: 527/30 nm), and Y5 (Ex: 620/60, Em: 700/75 nm) filters.

### Quantification of Chromosomal Organization and Nucleolar Domains in Different Cell Cycle Phases

The nuclear features were quantified by using the Fiji software^38^ on the digital images of interphase cells that were processed by two-dimensional Immuno-FISH for the codetection of chromosomes 3 and 6 together with the Ki67 and alpha-tubulin proteins. The measured parameters included the nuclear area and center of mass, lengths of the major and minor nuclear axes, mean intensities of nuclear staining and Ki67 immunoreactivity, the centromeric distance between the homologous chromosomes, the radial distance of chromosomes from the nuclear center, as well as the Ki67-intensity that colocalized with chromosomes 3 and 6. To avoid biases in nuclear orientation, cells with an oval nuclear morphology were included in the analysis, based on the ratio of the major (longitudinal) to minor (transverse) nuclear axis (aspect ratio) with a minimum value of 1.1. The nuclear orientation was further verified by the position of the nucleus with respect to the cytoplasm as reflected by the alpha-tubulin expression. Cells with a rounder nucleus were excluded considering the possibility that these samples may have had oval nuclei that landed at a more perpendicular angle on the minor axis during the cytocentrifugation on to the microscope slide.

Sorting the cells into different cell cycle stages was performed based on the DNA content that was calculated from the integrated density (area × intensity) of the nuclei as described.^41^ For the evaluation of nuclear parameters in the G1 phase, cells that were diploid for chromosomes 3 and 6 were considered, whereas the analysis in the G2 phase was conducted on the cells that were tetraploid for these chromosomes. Regarding the S phase, cells with an intermediate copy number of chromosomes 3 and 6 were included in the analysis.

### Quantification of Chromosomal Alignment and Missegregation in the Mitotic Cells

Images of cells that were enriched in different mitotic phases were evaluated by using the Fiji software.^38^ The mitotic cells were processed by two-dimensional Immuno-FISH for the co-detection of chromosomes 3 and 6 together with the alpha-tubulin protein. The expression pattern of alpha-tubulin facilitated the identification of distinct mitotic stages.^42^ Quantifications were restricted to the mitotic cells that were tetraploid for chromosomes 3 and 6. The prometaphase rosettes were identified by the arrangement of chromosomes in a wheel-like pattern around a chromatin-free center that was occupied by a star-shaped network of microtubule arms.^42^^,^^43^ The parameters that were quantified in the prometaphase rosettes included the angle between the homologous chromosomes with respect to the center and the centromeric distance between the homologous chromosomes that was normalized with respect to the mean length of nuclear axes. The criteria for missegregation included the presence of chromosomes 3 or 6 in micronuclei during any mitotic phase, asymmetrical/polarized distribution of homologous chromosomes from late anaphase onwards, and lagging chromosomes or bridges during telophase or cytokinesis.^44^^,^^45^ A minimum of 20 rosettes or mitotic cells per group and cell type were quantified in each experiment.

### Quantification of Chromosomal Missegregation in the Interphase Cells After Prolonged Incubation Under Normo- Versus Hyperglycemia

The UM cells that were seeded in six-well plates were cultured under normo- versus hyperglycemia for seven days with the refreshment of test medium every two to three days and without any treatment for mitotic arrest. Cells were then processed as cytospins for two-dimensional Immuno-FISH as described above. The copy numbers of chromosomes 3 and 6 were evaluated on the fluorescence microscopy images of the cells by using the Fiji software.^38^ Quantifications were restricted to the non-overlapping nuclei with clear signal(s) for both chromosomes. The number of cells that were quantified per group in each experiment varied between 206 to 310.

### Statistical Analysis

Data were analyzed by using the NCSS statistical software (Version 2024; NCSS, Kaysville, UT, USA) under Windows 10. The correlation of continuous parameters was assessed by the Spearman test. The normality of distribution was evaluated by the Shapiro-Wilk test. The equality of variances was examined by the Variance-Ratio and Modified-Levene tests. The association of binary factors with continuous variables was analyzed by the non-paired two-sided t-test assuming equal variance or the Mann-Whitney U test with normal approximation. The proportions were evaluated by Fisher's exact test or Pearson's χ^2^ test. All experiments were performed as three or four independent replicates using the individual UM cells or HTF, respectively. *P* values <0.05 were considered as significant.

## Results

### Characterization of the Primary Tumors That Were Used for the Generation of UM_f81 and UM_m60 Cells

The UM_f81 cells were isolated from a non-irradiated tumor with mixed cell morphology and moderate pigmentation whereas the UM_m60 cells originated from an irradiated sample with a predominantly spindle cell morphology and mild pigmentation. Both tissues exhibited BAP1-immunoreactivity in the nucleus and/or cytoplasm with intratumoral heterogeneity (Supplementary Fig. S1a). The UM_f81 and UM_m60 cells, as well as the 92.1 and OMM2.5 cell lines were positive for the melanoma markers MelanA and MITF and negative for the fibroblast specific protein 1 (Supplementary Fig. S1b).

### Enlargement of Nucleoli During Early Interphase in Multiple UM Cells Under Hyperglycemia

**Figure 1.**
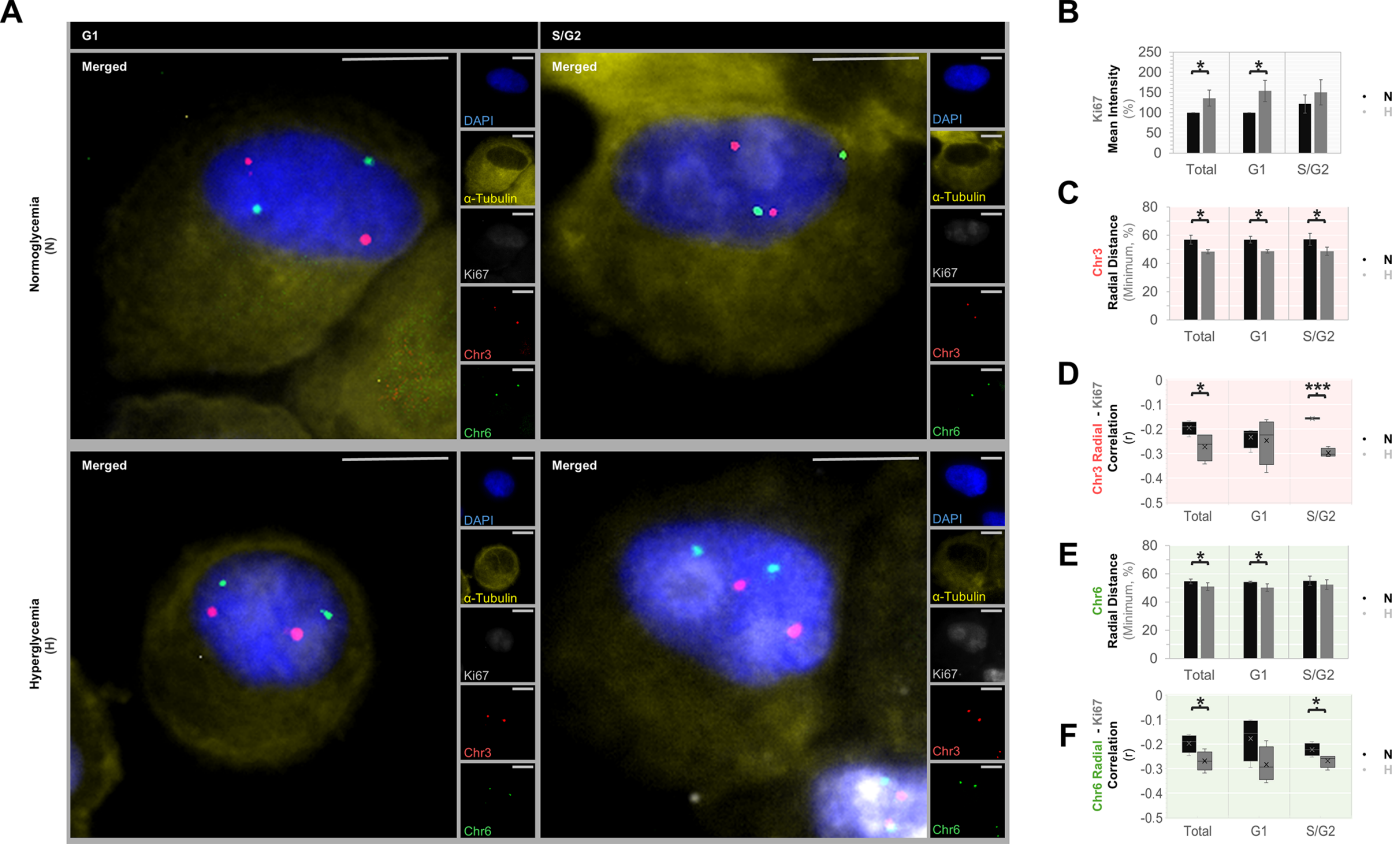
Centromeric positions of chromosomes 3 and 6 in the UM cells during interphase. **(A)** Co-detection of chromosome 3 and 6 centromeres (*red* and *green*, respectively) with the Ki67 (*gray*) and alpha-tubulin proteins (*yellow*) by two-dimensional Immuno-fluorescent in situ hybridization. The nuclei were counterstained in blue with DAPI. The alpha-tubulin stainings demonstrated the similar orientation of the nucleus with respect to the cytoplasm among the different groups. *Scale bars*: 10 µm. Quantification of the **(B)** mean Ki67 intensity, **(C)** minimum radial distance of chromosome 3, **(D)** correlation of the radial distance of chromosome 3 with the Ki67 intensity that colocalized with each centromere, **(E)** minimum radial distance of chromosome 6, and **(F)** correlation of the radial distance of chromosome 6 with the Ki67 intensity that colocalized with each centromere in the pooled UM cells from four donors. For each cell line, at least 50 cells with normal copy numbers of chromosomes 3 and 6 were quantified per cell cycle phase and treatment group. The mean values of two independent experiments were calculated for each cell line and used in the pooled quantifications. *P < 0.05, Mann-Whitney U test in **(B), (C),** and **(E)**. *P < 0.05, ***P < 0.001, two-sided t-test with equal variance in **(D)** and **(F)**.

Culturing the UM cells under hyperglycemia for one day resulted in the upregulation of the nucleolar protein Ki67, with a 1.4-fold increase in the mean nuclear Ki67 intensity compared to the cells that were maintained in normoglycemic medium (*P* = 0.01, Figs. 1A, 1B). We then evaluated whether this effect was due to the accumulation of more proliferative cells under hyperglycemia via the faster progression of UM cells into mitosis or due to the (hyper)activation of nucleoli regardless of the cell cycle phase. For this purpose, we classified the cells based on the cell cycle stages and compared the Ki67 expression further among the subgroups. Already during the G1 phase, the Ki67 protein became upregulated in the UM cells under hyperglycemia, with a 1.5-fold increase in the mean Ki67-intensity (*P* = 0.01). Although a similar pattern of Ki67-upregulation under hyperglycemia was observed in the individual UM cells during the S/G2 phases (data not shown), this effect did not reach significance when the cells from different donors were pooled (*P* = 0.25, Figs. 1A, 1B). These findings therefore demonstrated the hyperactivation of nucleoli in the UM cells under hyperglycemia already during the early interphase, resulting in the occupation of nucleus by more prominent nucleolar domains (Figs. 1A, 1B).

Hyperglycemia also resulted in a minor decrease in nuclear BAP1 immunoreactivity in the 92.1 and OMM2.5 cells under interphase, with an average reduction by 16% to 18% in the integrated density and mean intensity, respectively, of both cell lines, which failed to reach significance (Supplementary Fig. S2). The influence of hyperglycemia on BAP1 expression in the UM_f81 and UM_m60 cells could not be analyzed because of insufficient cellular material.

### Dislocation of Chromosome 3, and to a Lesser Extent Chromosome 6, Toward the Nuclear Center During Interphase in Multiple UM Cells Under Hyperglycemia

We next evaluated the positions of chromosomes 3 and 6 with respect to the nuclear center and nucleolar domains in the non-enriched UM cells that were cultured under normo- versus hyperglycemia for one day. In the normoglycemic UM cells, the centromeres of chromosome 3 were usually located at the nuclear periphery in opposing poles during interphase. In contrast, hyperglycemia resulted in the dislocation of at least one copy of chromosome 3 toward the nuclear center, decreasing the minimum radial distance by 15% (*P* = 0.02; Figs. 1A, 1C) and bringing the centromeres of chromosome 3 closer to each other (mean ± *SD* of normalized centromeric distance: 48.8% ± 2.5% vs. 54.7% ± 3.3% in the hyper- versus normoglycemic cells during the entire interphase, *P* = 0.04). The dislocated, more central copy of chromosome 3 was also usually colocalized with stronger nucleolar domains as demonstrated by the negative correlation of the radial distance of chromosome 3 with the Ki67 intensity at the respective centromeric position (Figs. 1A, 1D). Hyperglycemia also induced a milder decrease of approximately 7% in the radial distance of chromosome 6 centromeres particularly during the G1 phase (*P* < 0.05; Figs. 1A, 1E). The radial distance of chromosome 6 centromeres was negatively correlated with the colocalized Ki67 intensity mainly during the S/G2 phases (*P* < 0.05; Figs. 1A, 1F).

### Maintenance of Chromosome 3 Centromeres in Closer Proximity During Prometaphase in Multiple UM Cells Under Hyperglycemia

In mitotic cells, the alignment of condensed chromosomes presumably starts early during prometaphase, when the chromosomes become radially oriented in a wheel-like pattern defined as “rosettes,” with the centromeres facing the center and the microtubules being arranged like a star also in the center.^42^^,^^43^ We therefore evaluated the positions of chromosomes 3 and 6 in the prometaphase rosettes of UM cells that were cultured under normo- versus hyperglycemia for one day.

**Figure 2.**
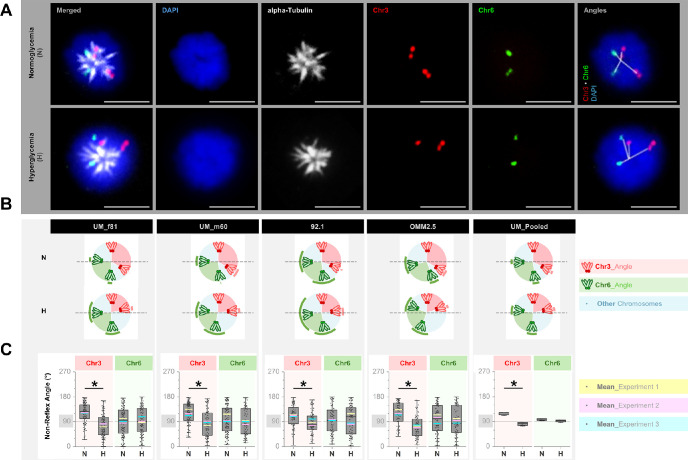
Centromeric positions of chromosomes 3 and 6 in the prometaphase rosettes of UM cells. **(A)** Co-detection of chromosome 3 and 6 centromeres (*red* and *green*, respectively) with the alpha-tubulin protein (*gray*) by two-dimensional Immuno-fluorescent in situ hybridization. The nuclei were counterstained in *blue* with DAPI. The *rightmost panels* demonstrate the angular positions of the centromeres of chromosomes 3 and 6 with respect to the rosette center. *Scale bars*: 10 µm. **(B)** Quantification of the mean angular positions of chromosomes (Chr) 3 and 6 in the prometaphase rosettes of individual UM cell lines and the pooled group. The arcs demonstrate the ± standard deviations from three independent experiments. The dashed lines indicate a theoretical alignment plane. Under normoglycemia (*N*), each half of the rosette appeared likely to receive one duplicated set of chromosome 3, whereas hyperglycemia (*H*) promoted the unequal distribution of chromosome 3 sets. The alignment of chromosome 6 copies was not notably altered under normo- versus hyperglycemia. **(D)** Box- and dot-plots demonstrating the non-reflex angles between the centromeres of chromosomes 3 versus 6 under normo- and hyperglycemia from three independent experiments. A minimum of 20 rosettes were quantified per group in each experiment. The mean angles in each experiment are highlighted with the colored lines. *P < 0.05, Mann-Whitney U test.

Under normoglycemia, the average angle between the centromeres of chromosome 3 varied between 112.0°–122.9° in the rosettes of UM cells from four donors, with a mean ± *SD* of 119.7° ± 5.1° in the pooled group. In contrast, the average centromeric angle between the chromosome 3 copies was significantly reduced below 90° under hyperglycemia in the UM rosettes of multiple donors, with a mean ± *SD* of 81.4° ± 6.3° and range of 73.8°–87.8° (*P* = 0.02, Fig. 2). Likewise, the normalized distance between the centromeres of chromosome 3 was reduced from 43.7% ± 4.0% to 32.8% ± 2.2% under normo- versus hyperglycemia, respectively (mean ± *SD* of the average distance from three independent experiments in each UM cell, *P* = 0.02). In contrast, the mean angle and distance between the centromeres of chromosome 6 were not significantly altered in response to hyperglycemia in the UM rosettes of four donors (Fig. 2).

### Hyperglycemia-Dependent Missegregation of Chromosome 3 Rather Than Chromosome 6 in the Mitotic UM Cells From Different Donors

**Figure 3.**
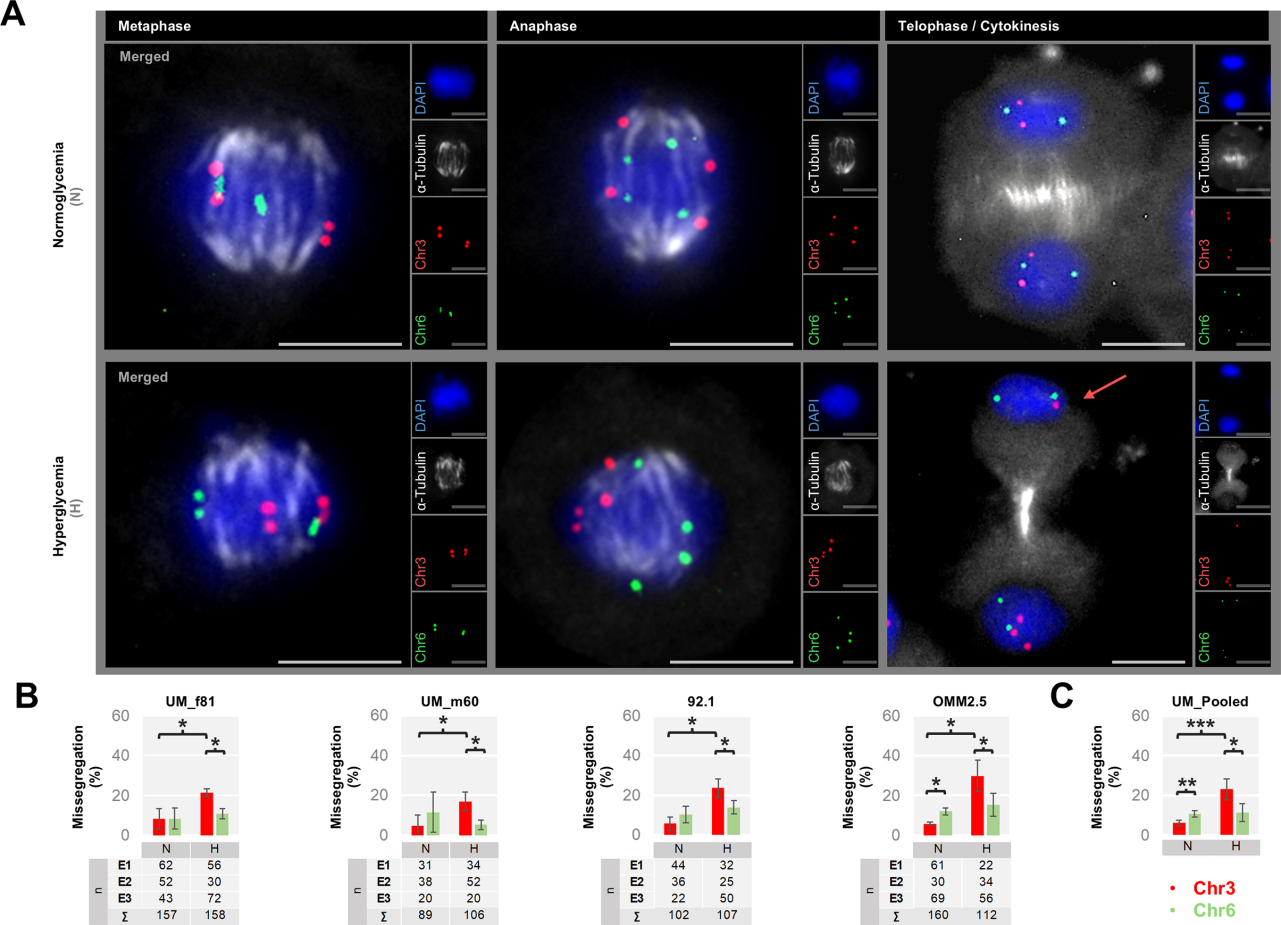
Alignment and segregation of chromosomes 3 and 6 in the uveal melanoma (UM) cells undergoing different mitotic stages. **(A)** Co-detection of chromosome 3 and 6 centromeres (*red* and *green*, respectively) with the alpha-tubulin protein (*gray*) by two-dimensional Immuno-fluorescent in situ hybridization. The nuclei were counterstained in *blue* with DAPI. *Arrow* indicates a daughter cell with monosomy 3. *Scale bars*: 10 µm. **(B)** Quantification of the missegregation rates of chromosomes (Chr) 3 and 6 in the individual UM cells under normo- (N) versus hyperglycemia (H) from three independent experiments. The number (*n*) of mitotic cells that were quantified in each group per experiment (E1–E3) is indicated in the tables underneath the bar plots. *P < 0.05, Mann-Whitney U test. **(C)** Pooled missegregation rates of chromosomes 3 and 6 calculated from the average missegregation rates of four UM cell lines. *P < 0.05, **P < 0.01, ***P < 0.001, two-sided *t*-test with equal variance.

During metaphase, when the chromosomes are tightly aligned along a narrow plate across the nuclear center,^42^ the centromeres of chromosome 3 remained closer to each other in the majority of UM cells under hyperglycemia compared to the normoglycemic cells (Fig. 3A). During anaphase, when the sister chromatids start to get pulled towards the opposing nuclear poles,^42^ hyperglycemia frequently resulted in the asymmetrical distribution of chromosome 3, with a single copy of chromosome 3 in one pole and three copies in the reverse pole. The polarized segregation of chromosome 3 under hyperglycemia persisted in the later mitotic phases (Fig. 3A). In addition, chromosome 3 tended to reside more often in micronuclei during any mitotic phase in the UM cells under hyper- versus normoglycemia (1.2% vs. 0.2% among the pooled group, respectively; *P* < 0.05, Pearson's χ^2^ test, images not shown). Accordingly, we observed a 3.8-fold increase in the percentage of UM cells with the missegregation of chromosome 3 (mean ± *SD* of missegregation rate: 6.1% ± 1.5% vs. 23.0% ± 5.3% in the normo- versus hyperglycemic UM cells from four donors, *P* < 0.001, Figs. 3A–C). In other words, hyperglycemia could significantly promote both the evident and potential development of monosomy 3 in the UM cells as detected by the uneven distribution of chromosome 3 in the clearly or incompletely divided nuclei during telophase/cytokinesis or the earlier mitotic stages, respectively (Figs. 3A–C). In contrast, the missegregation rate of chromosome 6 did not differ significantly, affecting an average of 10.5% to 11.3% UM cells under normo- versus hyperglycemia, respectively (*P* = 0.76, Figs. 3A–C). However, chromosome 6 rather than chromosome 3 was more prone to missegregation in the pooled UM cells under normoglycemia, with a 1.7-fold higher aneuploidy rate (*P* = 0.01, Fig. 3C). The number and percentage of UM cells with the missegregation of chromosome 3 or chromosome 6 in each cell type and experiment are presented in Table. Coexistence of monosomy 3 and chromosome 6 gain in the same nuclear pole during late mitosis was observed in only one of a total of 483 UM cells (0.2%) that were incubated under hyperglycemia but in none of the normoglycemic UM cells (*n* = 508) undergoing mitosis (*P* = 0.49, Fisher's exact test; image not shown).

**Table.** Number and Percentage of Cells With the Missegregation of Chromosome(Chr) 3 and/or Chr6 Under Normo-(N) Versus Hyperglycemia (H)

				Missegregation				
Cell	Treatment	Experiment	Total	Chr3, n (%)	Chr6, n (%)	*P* (Chr3 vs. Chr6)	*P* (Chr3_N vs. H)	*P* (Chr6_N vs. H)
UM_f81	N	1	62	3 (4.8%)	7 (11.3%)	0.32	**0.01**	1.00
		2	52	3 (5.8%)	6 (11.5%)	0.49	**0.03**	1.00
		3	43	6 (14.0%)	1 (2.3%)	0.11	0.61	0.25
		Sum	157	12 (7.6%)	14 (8.9%)	0.84	**<0.01**	0.85
	H	1	56	12 (21.4%)	6 (10.7%)	0.20		
		2	30	7 (23.3%)	4 (13.3%)	0.51		
		3	72	14 (19.4%)	6 (8.3%)	0.09		
		Sum	158	33 (20.9%)	16 (10.1%)	**0.01**		
UM_m60	N	1	31	1 (3.2%)	5 (16.1%)	0.20	0.36	0.10
		2	38	4 (10.5%)	7 (18.4%)	0.52	0.38	0.19
		3	20	0 (0.0%)	0 (0.0%)	1.00	0.11	1.00
		Sum	89	5 (5.6%)	12 (13.5%)	0.12	**0.01**	0.08
	H	1	34	4 (11.8%)	1 (2.9%)	0.36		
		2	52	10 (19.2%)	4 (7.7%)	0.15		
		3	20	4 (20.0%)	1 (5.0%)	0.34		
		Sum	106	18 (17.0%)	6 (5.7%)	**0.02**		
92.1	N	1	44	1 (2.3%)	5 (11.4%)	0.20	**0.04**	0.73
		2	36	2 (5.6%)	2 (5.6%)	1.00	**0.03**	0.22
		3	22	2 (9.1%)	3 (13.6%)	1.00	0.20	0.69
		Sum	102	5 (4.9%)	10 (9.8%)	0.28	**<0.001**	0.52
	H	1	32	6(18.8%)	5 (15.6%)	1.00		
		2	25	7 (28.0%)	4 (16.0%)	0.50		
		3	50	12 (24.0%)	5 (10.0%)	0.11		
		Sum	107	25 (23.4%)	14 (13.1%)	0.08		
OMM2.5	N	1	61	3 (4.9%)	8 (13.1%)	0.20	**0.03**	1.00
		2	30	2 (6.7%)	3 (10.0%)	1.00	**<0.01**	0.31
		3	69	4 (5.8%)	9 (13.0%)	0.24	**<0.01**	0.80
		Sum	160	9 (5.6%)	20 (12.5%)	**0.05**	**<0.001**	0.48
	H	1	22	5 (22.7%)	2 (9.1%)	0.41		
		2	34	13 (38.2%)	7 (20.6%)	0.18		
		3	56	16 (28.6%)	9 (16.1%)	0.17		
		Sum	112	34 (30.4%)	18 (16.1%)	0.02		
UM_pooled	N	Sum	508	31 (6.1%)	56 (11.0%)	**0.01**	**<0.001**	1.00
	H	Sum	483	110 (22.8%)	54 (11.2%)	**<0.001**		
HTF	N	1	163	12 (7.4%)	13 (8.0%)	1.00	0.41	**<0.01**
		2	193	28 (14.5%)	31 (16.1%)	0.78	0.57	**0.02**
		3	21	3 (14.3%)	2 (9.5%)	1.00	1.00	0.50
		4	62	5 (8.1%)	2 (3.2%)	0.44	0.52	**0.02**
		Sum	439	48 (10.9%)	48 (10.9%)	1.00	0.21	**<0.001**
	H	1	135	14 (10.4%)	28 (20.7%)	**0.03**		
		2	179	30 (16.8%)	48 (26.8%)	**0.03**		
		3	58	8 (13.8%)	10 (17.2%)	0.80		
		4	44	6 (13.6%)	8 (18.2%)	0.77		
		Sum	416	58 (13.9%)	94 (22.6%)	**<0.01**		

HTF, Human Tenon fibroblasts; n, Number; UM, Uveal melanoma; vs, Versus.

*P* values determined by Fisher's exact test. *P* < 0.05 are highlighted in bold.

### Increased Rate of Monosomy 3 in the UM Cells From Different Donors After Prolonged Incubation Under Hyperglycemia

To evaluate the long-term effects of normo- versus hyperglycemia on chromosome-specific aneuploidies, we also analyzed the missegregation rates of chromosomes 3 and 6 in the four UM cell lines after culturing for one week. The analysis was restricted to the interphase nuclei with clear signal(s) for both chromosomes. The cells were initially classified based on the copy number of either chromosome (defined as monosomy, disomy, or gain), which yielded a total of nine subgroups. In the “gain” subgroup, no distinction was made between the cells with three or more copies of chromosome 3 or 6.

**Figure 4.**
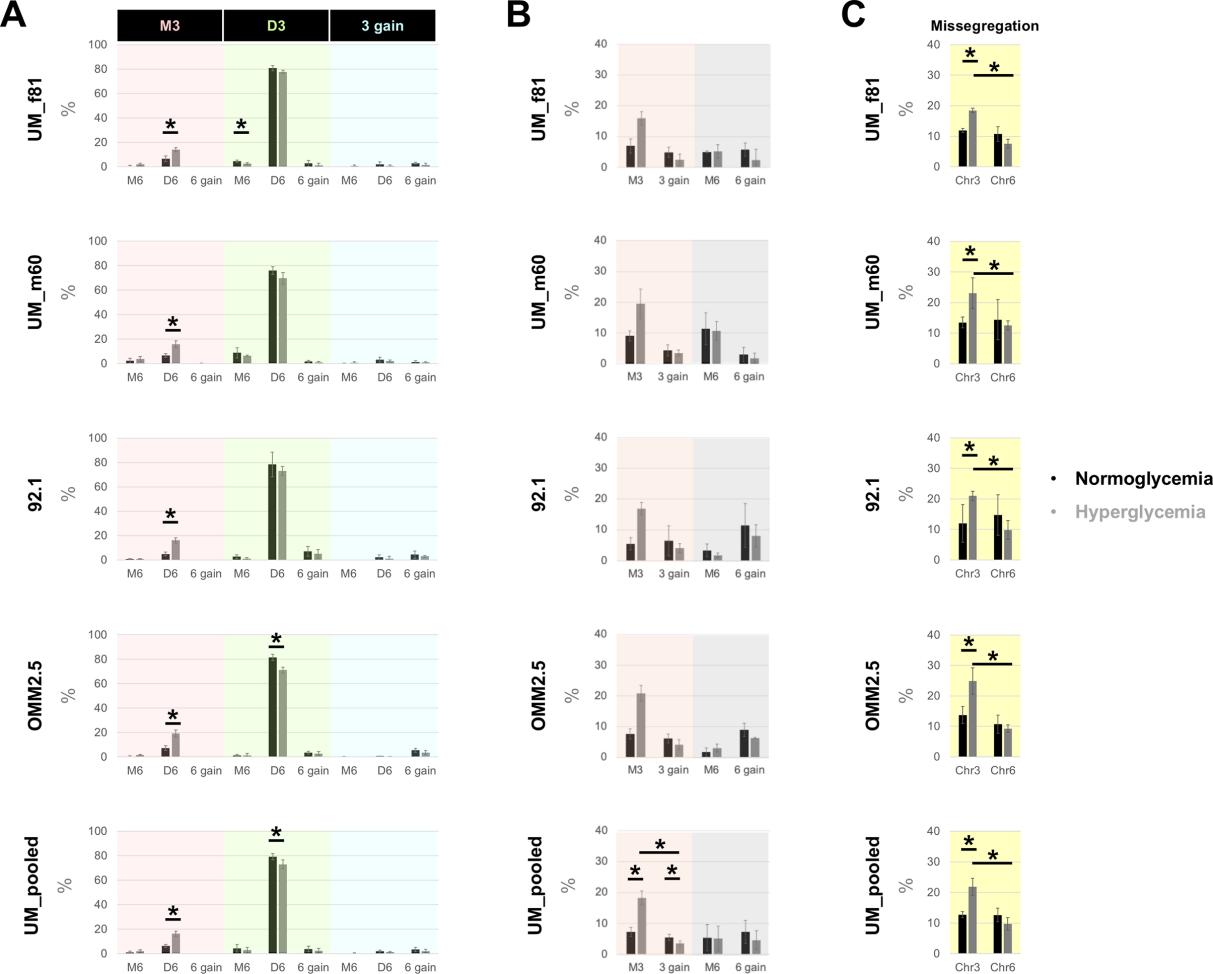
Missegregation rates of chromosomes 3 and 6 in different UM cells during interphase after seven days under normo- versus hyperglycemia. The bar plots demonstrate the percentages of cells that were classified based on **(A)** the copy numbers of chromosomes (Chr) 3 and 6, **(B)** monosomy versus gain of Chr3 or Chr6, and **(C)** the collective missegregation rate (monosomy and gain) of Chr3 and Chr6. Data represent the mean ± SD of *n* = 3 independent experiments with the individual cell lines and mean ± SD of the average percentage of the four cell types in the UM-pooled group. The number of cells that were quantified in each experiment and treatment group varied between 206–310. The total number of cells that were quantified in three independent experiments amounted to 2801 and 2858 under normo- versus hyperglycemia. *D*, disomy; *M*, monosomy. *P < 0.05 (Mann-Whitney U test).

Although the majority of the UM cells exhibited disomy 3 and 6 under both culture conditions, the incidence of this genotype was 6% lower in the cells that were grown under prolonged hyper- versus normoglycemia (mean ± *SD* 72.9 ± 3.6 vs. 79.1% ± 2.5%, respectively, in the pooled group; *P* < 0.05; Fig. 4A). In contrast, the rate of monosomy 3/disomy 6 was increased by at least twofold under hyperglycemia both among the individual cell lines and the pooled group (mean ± *SD* = 6.3% ± 1.0% vs. 16.3% ± 2.1% under normo- versus hyperglycemia, respectively, in UM_pooled; *P* < 0.05; Fig. 4A). The coexistence of monosomy 3 and 6 gain was detected in only one cell (UM_m60) under long-term normoglycemia and in none of the remaining cells (0.1% vs. 0% in a total of 678 and 692 UM_m60 cells under normo- versus hyperglycemia, respectively, from three independent experiments; nonsignificant; Fisher's exact test; Fig. 4A).

When we compared the incidence of monosomy versus gain of either chromosome, we observed the balanced distribution of cells with chromosome 6 aneuploidies, which ranged between 5% to 7% under normo- and hyperglycemia in the pooled group (non-significant; Fig. 4B). However, the incidence of monosomy 3 was increased by 2.5-fold under hyper- versus normoglycemia (mean ± *SD* = 18.3% ± 2.2% vs. 7.3% ± 1.5%, respectively, in UM_pooled; *P* < 0.05; Fig. 4B). In contrast, the gain of chromosome 3 showed a mild but significant decline of 2% in the hyperglycemic cells (mean ± *SD* = 3.5 ± 0.8 in the pooled group; *P* < 0.05; Fig. 4B). Accordingly, the collective missegregation rate (monosomy and gain) of chromosome 3 was significantly increased by approximately 1.7-fold under hyper- versus normoglycemia both among the individual cell lines and the pooled group (*P* < 0.05; Fig. 4C). Likewise, chromosome 3 rather than 6 was more prone to missegregation after prolonged hyperglycemia in all the UM cell lines and the pooled group (mean ± *SD* = 21.8% ± 2.8% vs. 9.8% ± 2.1% missegregation rate, respectively, in UM_pooled; *P* < 0.05; Fig. 4C).

The extent of chromosome 3 missegregation was significantly increased by 2.1-fold from approximately 6% to 13% in the mitotic cells after one day versus the interphase cells after seven days of incubation under normoglycemia (*P* = 0.02 in UM_pooled; Mann-Whitney U test). No time-dependent difference was observed in the missegregation rate of chromosome 3 under hyperglycemia or chromosome 6 in either test group.

### Altered Territories and Increased Missegregation Rate of Chromosome 6 Rather Than Chromosome 3 in Tenon Fibroblasts Under Hyperglycemia

**Figure 5.**
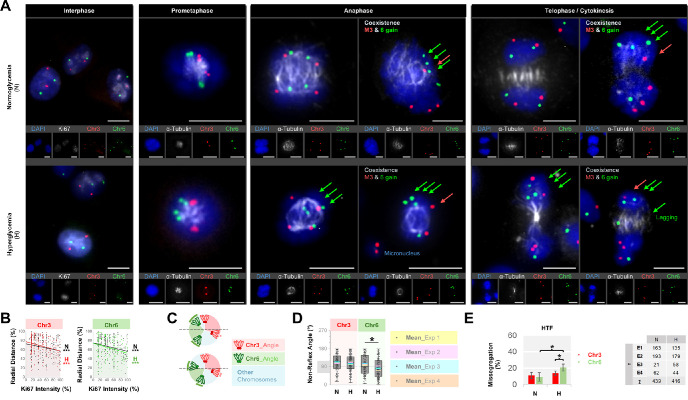
Centromeric positions of chromosomes 3 and 6 in the HTF during interphase and mitosis. **(A)** Co-detection of chromosome (Chr) 3 (*red*) and 6 centromeres (*green*) with the Ki67 or alpha-tubulin proteins (*gray*) during interphase or mitosis, respectively, by two-dimensional Immuno-fluorescent in situ hybridization. The *green* and *red arrows* indicate the gain of chromosome 6 and monosomy 3, respectively. *Scale bars*: 10 µm. **(B)** Correlation of the radial distances of chromosomes 3 and 6 with the Ki67 intensity at the respective centromeric positions during interphase. Quantifications were performed in at least 100 cells with normal copy numbers of chromosomes 3 and 6 in each group that were pooled from two representative experiments. ***P < 0.001, Spearman correlation. **(C)** Quantification of the mean angular positions of chromosomes 3 and 6 in the prometaphase rosettes of HTF. The *arcs* demonstrate the ± SD from four independent experiments. The *dashed lines* indicate a theoretical alignment plane. Under normoglycemia (*N*), each half of the rosette appeared likely to receive one duplicated set of chromosome 3 and 6, whereas hyperglycemia (*H*) induced a less favorable condition for the latter chromosome set by reducing the angle between the duplicated chromosome 6 pair. **(D)** Box- and dot-plots demonstrating the non-reflex angles between the centromeres of chromosomes 3 versus 6 under normo- and hyperglycemia from four independent experiments. A minimum of 20 rosettes were quantified per group in each experiment. The mean angles in each experiment are highlighted with the colored lines. *P < 0.05, Mann-Whitney U test. **(E)** Missegregation rates of chromosomes 3 and 6 in the HTF under normo- versus hyperglycemia from four independent experiments. The number (n) of mitotic cells that were quantified in each group per experiment (E1–E4) is indicated in the table on the right. *P < 0.05, two-sided *t*-test with equal variance.

To determine whether the hyperglycemia-induced alterations in chromosome territories are dependent on the cell type, we next evaluated the outcomes of hyperglycemia in human Tenon fibroblasts. Remarkably, the Tenon fibroblasts under hyperglycemia featured the exact opposite pattern compared to the UM cells, with the more prominent dislocation of chromosome 6 rather than chromosome 3 towards the nuclear center, resulting in a significant decline by 14% in the centromeric distance between chromosome 6 copies (mean ± *SD* = 58.8% ± 19.0% vs. 50.7% ± 18.9% in the normo- versus hyperglycemic fibroblasts from a representative experiment with n = 56–69 cells per group, respectively, *P* = 0.02, Fig. 5A). The dislocated centromeres of chromosomes 3 and 6 were usually colocalized with the activated nucleolar domains (Figs. 5A, 5B). Moreover, the angle between the centromeres of chromosome 6 was significantly reduced below 90° in the prometaphase rosettes under hyperglycemia, with a mean ± SD of 82.8° ± 9.2° versus 102.3° ± 5.2° in the hyper- versus normoglycemic fibroblasts (*P* = 0.01, *n* = 4 independent experiments, Figs. 5A, 5C, 5D). Accordingly, chromosome 6 was more prone to missegregation in the Tenon fibroblasts, with a 2.3-fold increase in its aneuploidy rate (mean ± SD of missegregation rate = 9.2% ± 5.3% vs. 20.7% ± 4.3% in the normo- versus hyperglycemic cells, *P* = 0.02, *n* = 4 independent experiments), whereas the missegregation of chromosome 3 was confined to 11.1% ± 3.9% to 13.6% ± 2.6% of the fibroblasts under normo- versus hyperglycemia (mean ± *SD* of *n* = 4 independent experiments; *P* = 0.31; Figs. 5A, 5E). Remarkably, some fibroblasts also exhibited the coexistence of monosomy 3 with the gain of chromosome 6 in the same daughter cell both under normo- and hyperglycemia (Fig. 5A). The number and percentage of Tenon fibroblasts with the missegregation of chromosome 3 and/or 6 in each experiment were presented in Table.

## Discussion

Despite the well-established association of monosomy 3 with a worse prognosis in UM,^1^^–^^5^ it has remained unknown why this fatal chromosomal anomaly occurs. Our findings provide the first evidence that hyperglycemia can favor the missegregation of chromosome 3 in UM cells in vitro. Under hyperglycemia, chromosome 3 tends to be at the wrong time in the wrong place.

The dislocation of chromosome 3 in response to hyperglycemia in the UM cells occurred early during interphase. The dislocated, more central copy of chromosome 3 was frequently colocalized with the activated nucleolar domains, as detected by the upregulation of the nucleolar protein Ki67 during the G1 phase.

Chromosomes with active genes relocate from the periphery toward the center to interact with the transcriptional machinery.^11^ Dislocation of chromosome 3 toward the center during interphase suggests that hyperglycemia upregulates nucleolar or non-nucleolar genes on chromosome 3 in UM cells. It has been shown that the short arm of chromosome 3 has a strong linkage to serum insulin concentrations and fasting insulin resistance index.^46^ However, the genes that are directly involved in these metabolic events have not been identified yet.

During the first stage of mitosis (prophase), the nucleolar components do not disappear completely, but become redistributed mainly on the periphery of the condensed chromosomes.^47^ The nucleolar organization during interphase may therefore be exerting long-lasting effects on the consequent chromosome organization in the later cell cycle stages. Accordingly, we observed the persistence of hyperglycemia-dependent alterations in the chromosome territories as the cells progressed from the G1/S- and G2/M-checkpoints into prophase.

Herein, the organization of chromosomes during prometaphase may be one of the crucial factors that ensures the proper segregation of chromosomes in the subsequent mitotic phases. The prometaphase entails a brief period defined as the formation of rosettes, where the condensed chromosomes become aligned radially in a wheel-shaped pattern, with the centromeres facing the center.^42^^,^^43^ The microtubules in the rosettes are also organized in the center in a star-shaped conformation, which presumably facilitates the connection of a microtubule arm with each chromosome.^42^ Remarkably, the angle between the copies of homologous chromosomes was never found to be below 90° in an earlier study on the prometaphase rosettes of human fibroblasts.^43^ This conformation possibly enables the distribution of one copy of each chromosome set to either half of the rosette by preventing the homologous chromosomes from getting too close to each other.^43^ In our study, the angle between the centromeres of chromosome 3 was significantly reduced below 90° in the rosettes of UM cells under hyperglycemia. This conceivably influenced the asymmetrical segregation of chromosome 3 during the later mitotic phases. In contrast, the Tenon fibroblasts featured the reverse pattern, with chromosome 6 being more prone to missegregation under hyperglycemia. This was associated and probably related to the reduced angle between the chromosome 6 centromeres in prometaphase rosettes. This is the first evidence for the disrupted alignment of distinct chromosomes in the prometaphase rosettes of both the UM cells and Tenon fibroblasts under hyperglycemia. This crucial mechanism appears to promote chromosome-specific aneuploidies in a cell-type dependent manner.

Our results also demonstrated the persistent effects of hyperglycemia on the post-mitotic fate of UM cells, with a significant increase by 1.7-fold in the missegregation rate of chromosome 3 in the interphase cells after one week. However, the magnitude of this increase was lower compared to the 3.8-fold effect in the mitotic UM cells after one day of hyperglycemia, possibly due to the significant rise in the percentage of normoglycemic cells with chromosome 3 missegregation after seven days. A possible reason underlying this outcome may be related to the differences in mitotic duration under normo- versus hyperglycemia. As stated in our methods, the hyperglycemic UM cells could indeed recover faster from the mitotic arrest, possibly leaving a shorter time for the correction of unstable kinetochore-microtubule attachments during metaphase,^48^ which may have further contributed to the missegregation of chromosome 3 from late anaphase onward. In contrast, the prolonged arrest of normoglycemic UM cells at metaphase may have enabled the repair of some faulty attachments between chromosome 3 pairs and the spindle fibers, resulting in a lower missegregation rate after one day. Yet, the long-term culture of UM cells was performed without any mitotic arrest with reagents that disrupt the microtubules, leaving the cells in both treatment groups less time for the correction of unstable kinetochore-spindle connections. The stable rate of chromosome 3 missegregation in the hyperglycemic UM cells after one and seven days may in turn have arisen from the poor growth rate of monosomy 3 cells in culture.^30^^,^^49^ In addition, we could not assess whether some monosomy 3 cells in the hyperglycemia group acquired isodisomy 3 during the long-term UM cultures. Nevertheless, the incidence of monosomy 3 was significantly elevated by 2.5-fold in the prolonged cultures under hyper- versus normoglycemia, underlining the persistent influence of hyperglycemia on the generation of this fatal anomaly in UM.

The percentage of monosomy 3 cells was also higher compared to the incidence of chromosome 3 gain after long-term hyperglycemia, as opposed to the balanced distribution of UM cells with the loss or gain of chromosome 6 regardless of the glucose concentration. Though uncommon in primary tumors, the partial gain of chromosome 3 was indeed reported in cultured UM cells.^50^ As stated in the definition of “gain” criteria in our methods, we did not distinguish between the cells with three or more copies of a given chromosome. Some mitotic cells with a prolonged arrest during spindle assembly checkpoint can indeed prematurely exit mitosis and enter a new G1 cycle with a tetraploid nucleus.^48^ We could therefore not discriminate whether the UM cells with chromosome 3 gain represented the sister counterpart of the monosomy 3 cells or whether they included some polyploid cells that have prematurely exited mitosis. Future studies with longer incubation periods would provide valuable insight on the cell cycle durations and post-mitotic fate of UM cells under normo- versus hyperglycemia.

Our study has several limitations, such as the relatively low number of cells in prometaphase or later mitotic stages that could be enriched for statistical analysis. The UM cells are indeed notoriously difficult to propagate in culture.^6^^,^^30^ These cells may be reaching replicative senescence earlier, rendering it more challenging to obtain mitotic cells. Prometaphase rosettes are also very short-lived, lasting around five to ten minutes,^43^ which complicates their enrichment. The abovementioned landmark study on the prometaphase rosettes involved an average of approximately 28 rosettes (range 8–46 rosettes) that were pooled from two types of fibroblasts for the angular quantification of different chromosomes.^43^ In our study, we quantified at least 20 prometaphase rosettes or mitotic cells per group in each experiment. To enrich a higher amount of cells in mitosis, it would have been convenient to synchronize the cells with additional treatments such as the thymidine block^51^ prior to the mitotic arrest with nocodazole. However, thymidine block itself can induce replicative stress and promote aneuploidy in cultured cells.^51^ In our study, we therefore avoided the synchronization of cells with further treatments and exposed the cells to only one round of mitotic arrest with nocodazole, despite the lower yield of mitotic cells that can be collected with the latter approach. A further limitation of our study was the analysis of chromosomal angles in a 2D model of prometaphase rosettes. Although a 3D model would have provided a more accurate insight, the 2D models have also been used for the analysis of chromosome territories in several other studies, including the landmark report on the spatial separation of homologous chromosomes by at least 90° in prometaphase rosettes.^43^^,^^52^

Our short-term cultures were also restricted to the analysis of interphase or mitotic cells that were diploid or tetraploid, respectively, for chromosomes 3 and 6. However, the copy number alterations of other chromosomes, partial gains or losses, and the presence of point mutations were not evaluated in this study. We could also report the centromeric positions of chromosomes 3 and 6 instead of the whole chromosome territories. We preferred to focus on the centromeric locations because the centromeres serve as the point of attachment for the microtubules, which enable the segregation of sister chromatids during mitosis.^42^ The alteration of centromeric positions would therefore be a crucial factor that can result in the incorrect segregation of chromosomes. In our study, we could not detect the location of chromosome 6p either, because of difficulties in the procurement of the 6p probe. We could therefore estimate the gain or loss of the whole chromosome 6 but not its individual arms. Interestingly, the long arm of chromosome 6 is also frequently affected in UM, with the loss of 6q being associated with a worse prognosis.^4^ Accordingly, the presence of two or more whole copies of chromosome 6 can be regarded as a more benign genotype in UM. We therefore believe that our findings on the predominance of monosomy 3 versus the gain of chromosome 6 in the UM cells under hyperglycemia provide highly relevant insight into the malignant transformation of UMs.

It remains unknown whether the benign potential of 6p gain in UM is conferred by some tumor suppressor genes on this chromosome arm or whether it arises simply due to the exclusion of monosomy 3.^2^^,^^3^ The 6p gain is indeed associated with a worse prognosis in several other malignancies, such as the retinoblastoma, cutaneous melanoma, colorectal cancer, or hepatocellular carcinoma.^53^ It would be therefore worthwhile to determine whether some genes on chromosome 6p act protective in the context of UM in future studies. Considering the frequent copy number alterations of chromosome 6 in other cancer types as stated above, our findings may also provide novel insight for the chromosome-specific aneuploidies in such malignancies. The generation of certain anomalies in distinct tumors may be facilitated by the chromosome territories, which are specific for each cell type depending on the functional specialization^11^ and which may be rearranged in response to external factors such as hyperglycemia as demonstrated by our novel findings. Interestingly, hyperglycemia and insulin resistance are also recognized as risk factors for the initiation, progression, or metastasis of diverse cancers, including the colorectal, gastrointestinal, head and neck, lung, breast, endometrial, prostate tumors and lymphoma.^54^^–^^59^ It would be therefore very exciting to analyze the influence of hyperglycemia on the chromosome-specific aneuploidies that predominate in other tumors in future studies. Hyperglycemia and insulin resistance in mice were also positively associated with the mitotic kinase Aurora-A (AurkA),^60^ which is frequently overexpressed in tumors with chromosomal instability.^61^ The outcomes of hyperglycemia on the AurkA-mediated dysregulation of centrosome maturation, microtubule dynamics, and chromosome congression^61^ therefore emerge as further aspects that deserve future investigation.

Interestingly, a recent study on the early detection of UM within a diabetic screening program reported that the identified lesions were usually smaller (*P* < 0.05) and contained a lower percentage of cells with monosomy 3 (*P* = 0.14).^62^ These findings may initially appear contradictory to our results. The authors have speculated that they detected asymptomatic small lesions at a very early stage prior to the accumulation of malignant features.^62^ Moreover, 6% of the control patients in the referred study also presented with diabetes.^62^ The type of diabetes, which was not stated in the aforementioned study, may also be crucial: In a recent study, Type 2 but not Type 1 diabetes was reported to be associated with a higher risk of metastasis in a Swedish cohort of UM patients.^24^ The patients with Type 2 diabetes have a history of insulin resistance with reduced levels of the insulin-sensitizing hormone adiponectin,^63^ whereas the adiponectin levels in Type 1 diabetes patients are elevated and exceed the values in normal, glucose-tolerant controls.^64^ Interestingly, adiponectin can also exert anti-carcinogenic actions on diverse tumor cells^63^ and we have previously reported the direct antiproliferative effects of adiponectin on cultured UM cells with a significant decrease in nucleolar size.^34^ Moreover, the deficiency of serum adiponectin in UM patients was associated with the development of metastases.^21^ Therefore, even if both the Type 1 and Type 2 diabetes patients present with hyperglycemia, the overproduction of adiponectin under Type 1 diabetes (possibly as a compensation to enhance the insulin sensitivity) may be exerting a suppressive effect on the growth of UM cells and possibly the missegregation of chromosome 3 by preventing nucleolar enlargement, contributing to smaller lesions with a lower incidence of monosomy 3.

In conclusion, our findings highlight hyperglycemia as a crucial factor that favors the generation of the malignant monosomy 3 in UM by the alteration of chromosome territories. This may be highly relevant both for the initial small primary tumor as well as for the dormant micrometastases in the liver. The liver serves as the major glucose reserve in the body, whereas insulin resistance promotes the excessive release of glucose from the liver,^22^ creating a hyperglycemic microenvironment that may be gradually awakening the dormant micrometastases.^65^ This might explain why metastatic disease in UM patients generally starts in the liver.^1^^–^^5^ Based on these results, the modulation of hyperglycemia and insulin resistance by lifestyle changes or therapeutic interventions emerge as simple adjuvant approaches that deserve further investigation in UM patients. By preventing the continuous generation of UM cells with monosomy 3, as suggested by the acquisition of this anomaly during both the early and late tumor development,^6^^,^^7^ the risk of fatal metastatic disease may be reduced and the chance to survive this deadly cancer may be improved.

## Supplementary Material

Supplement 1
