# Supplementary material for: Missegregation of Chromosome 3 and Generation of Monosomy 3 in the Proliferating Uveal Melanoma Cells Under Hyperglycemia
Source: Invest Ophthalmol Vis Sci. 2025 Dec 2;66(15):10. doi: 10.1167/iovs.66.15.10 (PMC12697705; doi:10.1167/iovs.66.15.10)
Supplement: Supplement 1 [file iovs-66-15-10_s001.docx]

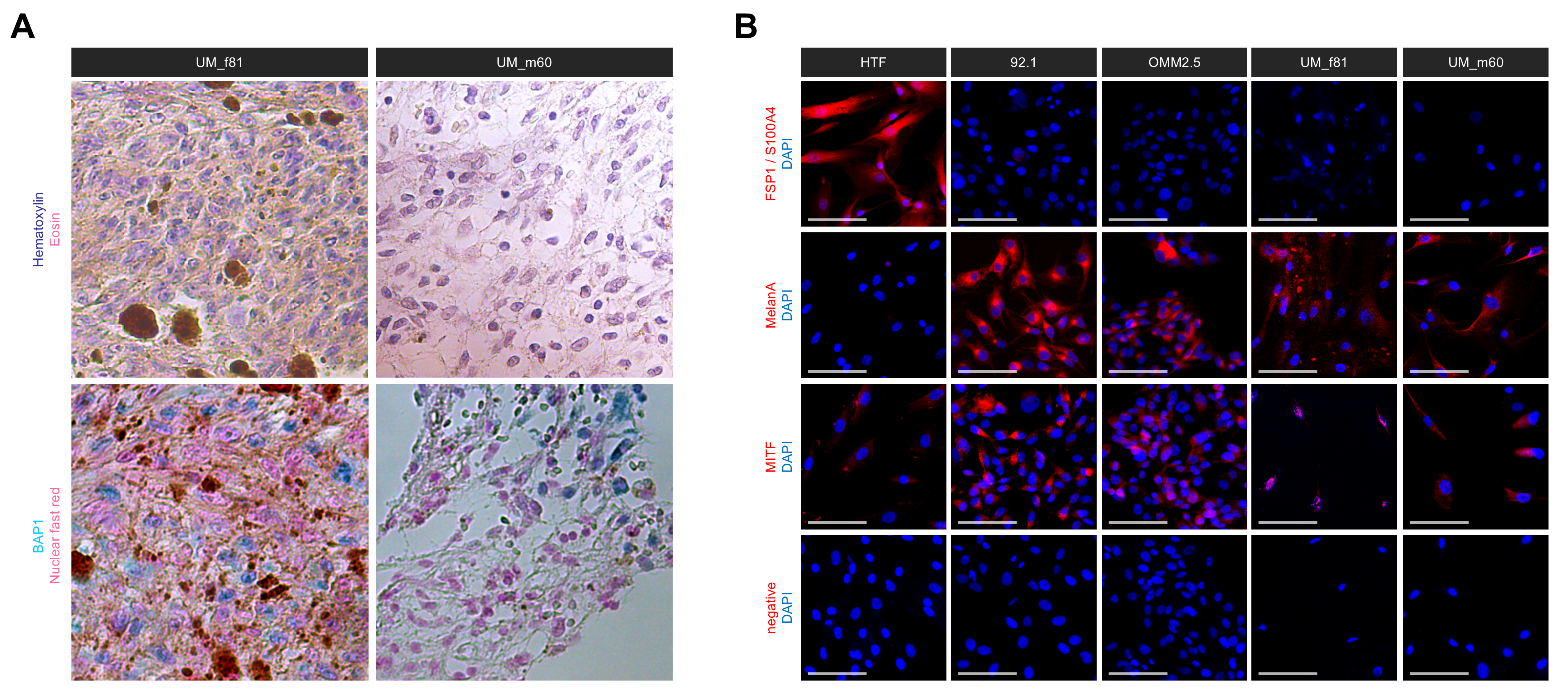


**Supplementary Figure S1.** Characterization of the primary uveal melanoma (UM) samples and cultured cells that were used in this study. (A) Hematoxylin-Eosin stainings and BAP1-Immunohistochemistry on the paraffin sections of the primary tumors that were used for generating the UM_f81 and UM_m60 cells. The UM_f81 cells were isolated from a non-irradiated tumor with a mixed cell morphology and moderate pigmentation (brown) whereas the UM_m60 cells originated from an irradiated sample with a predominantly spindle cell morphology and mild pigmentation. Both tissues exhibited BAP1-immunoreactivity with intratumoral heterogeneity. Magnification: 200X. (B) Fluorescent-immunocytochemistry for fibroblast- and melanocyte markers on the cultured human Tenon fibroblasts (HTF) and UM cells. The fibroblast specific protein 1 (FSP1), which is encoded by the S100A4 gene, is a highly sensitive marker for resting and activated fibroblasts.^1^ The MelanA antigen resides on a cytoplasmic protein that is involved in melanosome biogenesis and serves as a specific marker for melanocytic lineage.^2,3^ The microphthalmia-associated transcription factor, which is encoded by the MITF gene, is a regulator of melanocyte survival, development, and proliferation.^4^ Despite the sensitivity of MITF as a marker of melanocytic proliferations,^4^ the immunoreactivity for MITF has also been detected in several non-melanocytic cells including fibroblasts.^5^ The negative controls were performed by omitting the primary antibodies. Scale bars= 100 µm.

**
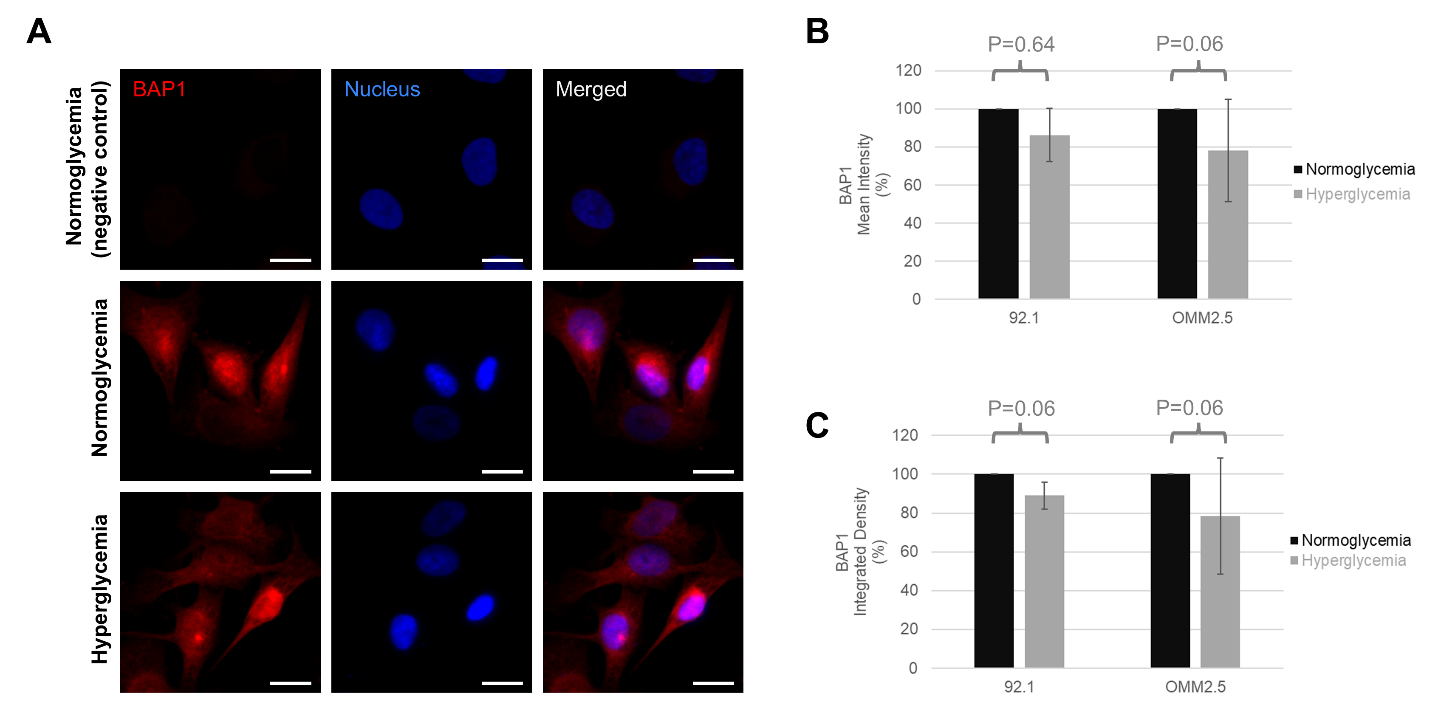
**

**Supplementary Figure S2.** BAP1 expression during interphase in the 92.1 and OMM2.5 cells under normo- versus hyperglycemia. (A) Representative images of the fluorescent immunocytochemistry for BAP1 (red). The nuclei were counterstained in blue with DAPI. The negative control was performed by omitting the primary BAP1 antibody. Scale bars= 20 µm. Quantification of the (B) mean intensity and (C) integrated density of the BAP1 immunoreactivity that were colocalized with the nuclei (mean ± standard deviation of n=3 independent experiments). The number of quantified cells per group in each experiment varied between n=403-616 and n=467-906 for the 92.1 and OMM2.5 cell lines, respectively. P values were determined by the Mann-Whitney U test.

**Supplementary References**

1. Sugimoto H, Mundel TM, Kieran MW, Kalluri R. Identification of fibroblast heterogeneity in the tumor microenvironment. *Cancer Biol Ther.* 2006;5(12):1640–1646.
2. De Mazière AM, Muehlethaler K, van Donselaar E, et al. The melanocytic protein Melan-A/MART-1 has a subcellular localization distinct from typical melanosomal proteins. *Traffic.* 2002;3(9):678–693.
3. Weinstein D, Leininger J, Hamby C, Safai B. Diagnostic and prognostic biomarkers in melanoma. *J Clin Aesthetic Dermatol.* 2014;7(6):13–24.
4. Goding CR, Arnheiter H. MITF-the first 25 years. *Genes Dev.* 2019;33(15-16):983–1007.
5. Mohanty SK, Sharma S, Pradhan D, et al. Microphthalmia-associated transcription factor (MiTF): promiscuous staining patterns in fibrohistiocytic lesions is a potential pitfall. *Pathol Res Pract.* 2018;214:821–825.
